# Professor Herrick

**Published:** 1847-06

**Authors:** 


ARTICLE X.

PROFESSOR HERRICK.

Our colleague who, our readers are aware, has been, during the past year, serving as surgeon in the army in Mexico, has returned to his post at home, and will hereafter devote himself to the practice of his profession in Chicago, to his labors as co-editor of the Journal, and to his duties as professor of anatomy in Rush Medical College. As he has seen much service in his department while absent, having been principal acting surgeon at the bloody conflict of Buena Vista, our readers have a right to expect, and we doubt not will shortly receive, a full account of all that is of interest pertaining to the profession, that came under his observation.

